# Integration and Validation of the Genome-Scale Metabolic Models of *Pichia pastoris*: A Comprehensive Update of Protein Glycosylation Pathways, Lipid and Energy Metabolism

**DOI:** 10.1371/journal.pone.0148031

**Published:** 2016-01-26

**Authors:** Màrius Tomàs-Gamisans, Pau Ferrer, Joan Albiol

**Affiliations:** Departament d'Enginyeria Química, Biològica i Ambiental, Universitat Autònoma de Barcelona, 08193 Bellaterra (Cerdanyola del Vallès), Barcelona, Spain; Universidade Federal de Vicosa, BRAZIL

## Abstract

**Motivation:**

Genome-scale metabolic models (GEMs) are tools that allow predicting a phenotype from a genotype under certain environmental conditions. GEMs have been developed in the last ten years for a broad range of organisms, and are used for multiple purposes such as discovering new properties of metabolic networks, predicting new targets for metabolic engineering, as well as optimizing the cultivation conditions for biochemicals or recombinant protein production. *Pichia pastoris* is one of the most widely used organisms for heterologous protein expression. There are different GEMs for this methylotrophic yeast of which the most relevant and complete in the published literature are iPP668, PpaMBEL1254 and iLC915. However, these three models differ regarding certain pathways, terminology for metabolites and reactions and annotations. Moreover, GEMs for some species are typically built based on the reconstructed models of related model organisms. In these cases, some organism-specific pathways could be missing or misrepresented.

**Results:**

In order to provide an updated and more comprehensive GEM for *P*. *pastoris*, we have reconstructed and validated a consensus model integrating and merging all three existing models. In this step a comprehensive review and integration of the metabolic pathways included in each one of these three versions was performed. In addition, the resulting iMT1026 model includes a new description of some metabolic processes. Particularly new information described in recently published literature is included, mainly related to fatty acid and sphingolipid metabolism, glycosylation and cell energetics. Finally the reconstructed model was tested and validated, by comparing the results of the simulations with available empirical physiological datasets results obtained from a wide range of experimental conditions, such as different carbon sources, distinct oxygen availability conditions, as well as producing of two different recombinant proteins. In these simulations, the iMT1026 model has shown a better performance than the previous existing models.

## Introduction

Genome-Scale metabolic models (GEMs) have become one of the most useful and widely employed tools in systems biology in the last fifteen years [1]. Since the first genome based metabolic model was presented [2], a huge number of models have been developed for a broad variety of species, from archaea and bacteria, to higher eukaryotes [3]. These models link genotype with phenotype; thus, they can predict the behavior of an organism under certain environmental conditions [4–6]. GEMs have been applied for both descriptive and predictive purposes. They have been used for multiple omics data integration [4, 7], for discovering metabolic network properties and organism capabilities as well as for comparing these capabilities between organisms. In addition they are commonly used for predicting metabolic engineering targets to improve growth and production of chemicals or recombinant proteins, thus making processes more efficient at industrial-scale [8–12].

Regarding the expression of heterologous proteins, *Pichia pastoris* has become more extensively used for industrial applications [13, 14]. It has many interesting properties that make it suitable for recombinant protein production. One of the main advantages is the availability of well-established protocols and techniques for its genetic manipulation [15, 16] that enables the expression and secretion of recombinant proteins, even those with complex post-translational modifications [17–19]. Moreover, there are a number of promoters available which are suitable for regulation with different carbon sources [20, 21]. Specifically, one of the most commonly used is the very powerful and tightly regulated *AOX* promoter, inducible with methanol [19]. Finally *P*. *pastoris* is able to grow up to high cell densities, resulting in high protein production yields [22, 23]. Due to the increasing interest in using this advantageous methylotrophic yeast, several efforts have provided tools to better understand its operation, from physiological characterization to metabolic modelling.

The first metabolic models for *P*. *pastoris* were limited to the central carbon metabolism and were used for ^13^C flux data analysis [24–27]. In 2009, the genome of two different strains, DSMZ 70382 [28] and GS115 [29], was sequenced. More recently, a third strain (CBS7435) was also sequenced and annotated [30] showing some discrepancies with previously reported sequences. Once the genomic data was available, two GEMs were published simultaneously: iPP668 [31] corresponding to GS115 strain, and PpaMBEL1254 [32] based on the DSMZ 70382 genome. Two years later, a third model (iLC915) became available, also based on the GS115 genome [33]. Moreover, other GEMs derived by automatic application of reconstruction algorithms are also available [34, 35] which will not be discussed in this paper due to their low level of curation and comprehensiveness.

*P*. *pastoris’* GEM models are potentially useful platforms for bioprocess design and optimization, as well as for strain metabolic engineering [36, 37]. In fact, Nocon and co-workers [38] have already used PpaMBEL1254 to predict overexpression and deletion mutants to enhance recombinant protein production.

Each one of the three currently available models is fully compartmentalized, but they differ in the number of reactions and metabolites. The first two models, iPP668 and PpaMBEL1254 have a comparable number of associated genes, reactions and metabolites, as well as similar metabolite and reaction identifications and nomenclature. On the other hand, iLC915 incorporates more *P*. *pastoris* specific gene-protein-reaction associations and hence, a larger number of genes, but several extracellular and nuclear reactions are missing. In general terms, these models cover the same metabolic processes, but iLC915 is more detailed. Nevertheless, there still exist some critical issues in these models, such as missing and divergent information or reactions that require manual revision and curation.

Similar to other organisms, such as *Saccharomyces cerevisiae*, new versions and updated models integrate previous versions and incorporate new features and information from newly published literature. In the case of *S*. *cerevisiae*, despite the existence of other versions, a consensus metabolic model was developed [39] and it was further developed and upgraded, being expanded and revised up to the 7^th^ version [40–43].

In this work, we compare the models of *P*. *pastoris* and provide an upgraded version. As mentioned above two different strains were used to obtain these models. Nonetheless, there is a high degree of identity at the amino acid coding sequences level (93.7%) and functional annotation between the two genome sequences [32]. In addition, no differences were observed in reactions involved in the metabolization of the different carbon sources [44]. Therefore, a major objective of our study was to obtain a general model that can be applicable to both strains. Furthermore, an extensive analysis was performed on several pathways, comparing the three models and updating them with the newly published literature. Recent findings on *P*. *pastoris* physiology and metabolism enabled to complete sphingolipid biosynthesis metabolism and glycosylation pathways, as well as the oxidative phosphorylation electron transport chain. Furthermore, we included different biomass compositions specific for each of the alternative carbon sources used. Finally, the model accuracy was tested in a variety of physiological conditions.

## Materials and Methods

### Model merging

For the model comparison, an initial step of metabolite nomenclature unification was required. In PpaMBEL1254 only the identifier (ID) was available in the SBML file, i.e. neither the complete name of metabolites nor any association to a reference database was included. These metabolite IDs, were mostly the standard IDs most commonly used and therefore also included in both in BiGG [45] and The SEED [46] reference databases. After this first metabolite parsing and renaming step was done, MetaNetX [47] database was used in order crosslink information from KEGG [48], ChEBI [49] and MetaCyc [50]. This step not only allowed unifying metabolite names but also to include its molecular formula and charge at pH 7.2.

Once all metabolite names were unified, PpaMBEL1254 and iPP668 were compared using ModelBorgifier [51], thereby obtaining a first pre-merged model. In a second step, this merged model was compared to iLC915, generating a first draft of the consensus model. Due to important differences in model structure between iLC915 and the other models, a manual comparison was necessary. This was performed by analyzing the structure of each of the remaining pathways or subsystems. Differences were resolved according to the available literature, comparing the reactions with those included in two latest versions of the consensus *S*. *cerevisiae* GEMs [42, 43] or in another recently published *S*. *cerevisiae* GEM [52]. Divergences in gene assignments were resolved using *P*. *pastoris* or *S*. *cerevisiae* literature. The *P*. *pastoris* high quality sequence annotation [30] and the automatic reconstructions for *P*. *pastoris* [34, 35] were also used to verify annotations and gene-reaction assignments from the previous models. Finally, pathway revamping and addition of new reactions was performed based on available yeast literature and metabolic pathways/reaction databases [45, 50, 53, 54].

Eventually, the network was loaded into a convenient environment for debugging [55]. Thus, both COBRA [56] and RAVEN [57] toolboxes were used in order to ensure pathway connectivity and biomass formation. Duplicated reactions in the final model were deleted and blocked reactions were connected (gap filling) to the network when few steps were required. In addition, the elemental mass balance of each reaction was checked and corrected when unbalanced. The final model (S1 and S5 Files) can also be obtained in SBML format from BIOMODELS database with accession number: MODEL1508040001 [58]. The SBML model was generated with the RAVEN toolbox [57] and validated with SBMLeditor [59].

### Biomass and recombinant protein composition

The biomass reaction is defined by the sum of biomass components, grouped in macromolecules (carbohydrates, proteins, lipids, DNA, RNA), essential cofactors and ATP consumption associated to growth. This equation was adapted depending on culture conditions or carbon source used in accordance to the available literature experimental data [60, 61]. In addition, composition of each macromolecule type, such as lipid and carbohydrate, was updated and extensively detailed due to the recently published detailed information of the specific composition [62, 63]. See S2 File for details in composition and calculations.

The model was also tested for the expression of two different recombinant proteins under different growth conditions: i) the antibody fragment 2F5 (FAB), expressed constitutively under the *GAP* promoter [64] and, ii) a *Rhizopus oryzae* lipase (ROL), regulated by the methanol inducible AOX promoter [65]. The dataset from the FAB-producing strain was used for simulations in oxygen limiting growth conditions [26, 66], whereas the dataset from the ROL producing strain was used in simulations for glycerol-methanol co-feeding experimental conditions [25, 61]. Reactions for heterologous protein production were included considering different levels: DNA sequence, transcription and mRNA formation, as well as translation and protein formation. Similarly to PpaMBEL1254 and iLC915, a ratio of 1:100:10^5^ between recombinant DNA (gene copies), mRNA and heterologous protein was assumed, as described in [32]. These equations also include energetic requirements for polymer formation [67]. Details of DNA, RNA and amino acid composition of each protein, as well as equations for the biosynthesis of their components can be found in S3 File.

### Energy requirements

Before the validation step, classic energetic parameters were estimated using experimental data. These parameters are the growth-associated maintenance energy (GAME) and non-growth-associated maintenance energy (NGAME). Both are represented as ATP consumption in the model. For the NGAME calculation, a classical approach was used [68]. For the glucose-limited cultivations, the glucose uptake rate (mmol glucose·gDCW^-1^·h^-1^) was represented against the specific growth rate using data from [24, 60, 69, 70]. The *y*-intercept of the linear regression line to this data corresponds to the amount of glucose needed for maintenance by non-growing cells. Using this value and the model, the NGAME can be calculated by maximization of the ATP turnover per mmol of glucose for the case of no biomass growth. Using the obtained value as fixed value for the non-growth associated maintenance, GAME is determined by adjusting the ATP consumption coefficient in biomass equation to fit biomass-substrate yields using experimental data (including CO_2_ and O_2_ constrains) from [60].

However, for the case of glycerol:methanol growth conditions NGAME was directly taken from the calculated values reported in [61]. This was necessary due to the range of cultivation conditions considered and the insufficient experimental data available. Using these values, and similarly to the glucose-only growth condition, the GAME values for glycerol:methanol conditions were calculated by fitting the predicted values to the range of experimental biomass-substrate yields reported in [61].

### Model analysis and validation

Model analysis and validation were performed using both RAVEN [57] and COBRA [56] toolboxes as described below.

Carbon assimilation capabilities were determined maximizing growth rate and arbitrarily constraining the carbon source influx to 10 mmol·gDCW^-1^·h^-1^ except were otherwise stated.

Reaction essentiality was determined performing an additional set of simulations. The procedure consisted on sequentially deleting each reaction of the model, maximizing biomass production and calculating the ratio of the resulting growth rate over the wild type result (GR_KO_ = growth_KO_/growth_WT_). The ratios obtained allowed to classify each reaction into three categories: i) essential (GR_KO_ = 0), ii) partially-essential (0 < GR_KO_ < 1) iii) non-essential (GR_KO_ = 1).

Evaluation of the effect of oxygen limiting conditions on glucose cultures was performed constraining glucose and oxygen uptake rates to the measured values [66]. For the glycerol:methanol experimental conditions, only glycerol and methanol uptake rates were constrained to the experimental values [24] while O_2_ uptake rate was left unconstrained. In all these cases biomass, CO_2_ and by-products were left as unconstrained positive values and therefore appeared as calculated products were necessary. In all these cases biomass production was the maximized objective function.

## Results and Discussion

### Model merging

**Fig 1:**
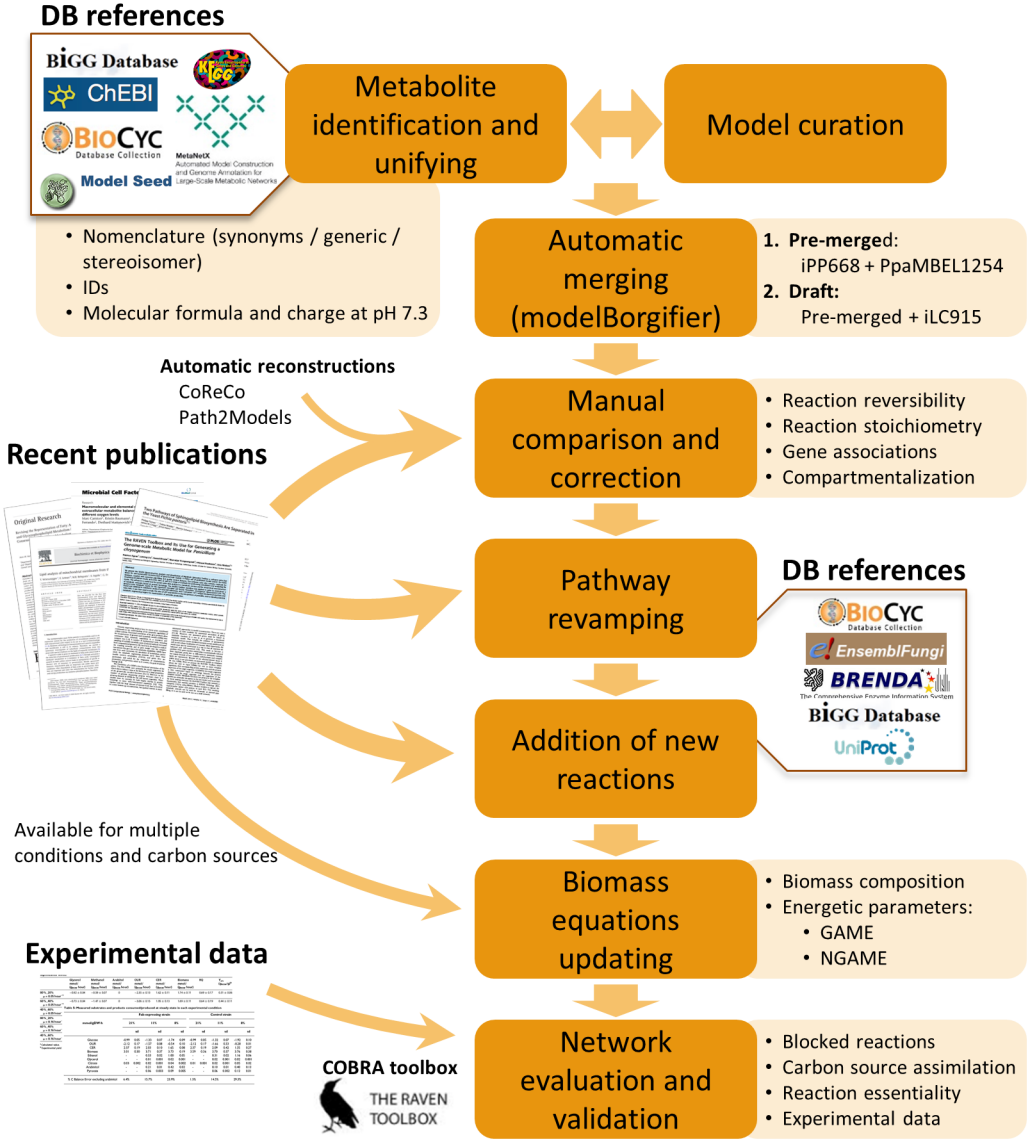
Schematic overview of the major steps involved in the construction of *P*. *pastoris* GEM iMT1026. The process of GEMs integration started with the metabolite identification, unifying nomenclature and curation steps of iPP668, PpaMBEL1254 and iLC915. The continuation steps were performed on the resulting pre-merged model and subsequent drafts. Experimental data for model validation was taken from [25, 60, 61, 66].

As described in Materials and Methods section and summarized in Fig 1, the generation of the new model consists of several steps. In the first step of reconciliation, PpaMBEL1254 and iPP668 were automatically compared using modelBorgifier [51]. After the initial pairing step, 75% of the complete set of reactions was identified as identical (exact coincidence) reactions. This pre-merged model was compared with iLC915 resulting in a low number of identical or equivalent reactions (36% of the complete set). Nevertheless, a larger number of reactions were comparable. Those mainly differ in having different stoichiometric coefficients, being assigned to different compartments, decomposed in multi-step reactions, or using alternative names for metabolites (different synonyms, generic names or corresponding to enantiomer compounds).

The iPP668 and PpaMBEL1254 models were the first models to be published, and they are more similar to each other than to iLC915. Approximately 83% of the reactions from PpaMBEL1254 are present in iPP668 and 89% of the reactions in iPP668 are shared with PpaMBEL1254. Overall, models iPP668 and PpaMBEL1254 have a 75% of reaction identity. Furthermore, similar nomenclature and abbreviations are used in these two models. In addition, model structure and detail are similar to iND750 [71], from *S*. *cerevisiae*, and those models in the BiGG database [45].

The third published model, iLC915, has many differences with the previous two models. Its nomenclature and structure is KEGG-based [48]. Therefore, its metabolites are fully protonated and many pathways include the same number of steps described in KEGG. That is, many condensed or simplified metabolic branches in the other two models appear decomposed as multi-step reactions in iLC915. Such reaction differences among the models are one of the major reasons for the low pairing of iLC915 with the other two models and seem to be the result of the main database or model scaffold used as basis for model reconstruction.

### Updated pathways

As a result of the model comparison and merging step, some divergences in several pathways of the three existing models were evident. In addition, some of them were incomplete or misrepresented in all three models. Therefore we engaged in a curation step according to the recently published literature and database information. Nevertheless, some difficulties in gene and pathway verification were found. The fact that new genomes are usually automatically annotated, at least in an initial step, and that enzyme activities or functions are inferred by homology, propagates errors from already annotated sequences to the new ones [72]. This issue arises when there is limited biological knowledge of the organism. Furthermore, not only genome annotations are based in other organisms and sequences, but GEMs are also commonly developed from previous existing models. As a result, annotation errors or misrepresented pathways are also spread to the subsequent new/updated models. Moreover, as new GEMs are mostly based in pre-existing reconstructions, few new metabolic reactions are often incorporated in the novel GEMs versions. Consequently, the metabolic potential and biological diversity is often not fully reflected in the GEMs and the total number of enzymatic activities present in the existing models remains far below from the complete catalog of enzymatic steps described in the literature for each organism [3].

In the case of *P*. *pastoris*, the current annotation of its genome is rather limited [73], with most of all annotations being inferred by homology, mainly from *S*. *cerevisiae*. According to the best annotation available *P*. *pastoris* has 5040 protein-coding annotated genes of which only 3532 has been assigned an Ontology term and all but 21 annotations are automatically inferred [73]. Despite to the fact that *P*. *pastoris* and *S*. *cerevisiae* belong to the same family (*Saccharomycetaceae*), they present significant differences in their metabolic capabilities. Thus, besides *P*. *pastoris* well known additional pathways such as the methanol incorporation steps, other significant metabolic differences exist. More specifically, in this work, pathways such as sphingolipid biosynthesis, oxidative phosphorylation and glycosylation pathway, were adapted and redefined, as described below.

### Fatty acid biosynthesis

Due to the limited information on specific fatty acid (FA) metabolic pathways in *P*. *pastoris*, it was assumed that most of *S*. *cerevisiae* fatty acid pathways were identical in the *P*. *pastoris* case. According to Hiltunen and co-workers [74], the latest version of yeast consensus model [43] and iTO977 model of *S*. *cerevisiae* [52], fatty acid biosynthesis takes place both in mitochondria and cytosol by fatty acid synthase (FAS) type II and I, respectively [75]. FAS type II has individual enzymes for each reaction in fatty acid *de novo* biosynthesis and elongation. Despite it is well known that mitochondrial FAS type II synthetizes at least up to C_8_ fatty acid, some evidences suggest that this system can synthesize longer fatty acids [74, 76]. While in *S*. *cerevisiae* 7^th^ version of the consensus model [43] mitochondrial biosynthesis is up to C_8_, this model also include reactions for up to C_18_ biosynthesis.

On the other hand, cytosolic FAS is a complex formed by Fas1p and Fas2p within which the successive elongation reactions take place and only the final acyl-CoA is released [77]. The final products of this cytosolic complex are considered to be C_14_ to C_18_ acyl-CoAs, mainly because they are the main fatty acids found in *P*. *pastoris* [62]. The biosynthesis takes place inside the complex in a number of four step cyclic reactions for each acetyl-CoA added. Different number of cycles results in a range FA (C_14_, C_16_ and C_18_ acyl-CoAs). In addition to *de novo* biosynthesis, *P*. *pastoris* also has fatty acid elongation enzymes, which are able to extend C_12-14_ fatty acids and generate very long chain fatty acids (up to C_26_).

Activation of free fatty acids (FFA) was considered to take place in cytosol only for C_14_, C_16_ and C_18_ FFA, as well as their respective acyl-CoA hydrolysis. Finally, only acyl-CoA desaturations were included (that is not acyl-ACP or FFA) according to the pathway defined in *S*. *cerevisiae* [43].

### Fatty acid oxidation

Two different transport mechanisms are commonly described depending on the FA chain length [78–80], both being closely coupled to its activation to acyl-CoA [81–85]: on the one hand, a simple diffusion and further activation of medium-chain fatty acids (up to C_12_ chain length) and, on the other hand, long and very long chain fatty acids are translocated as acyl-CoA concomitant with the corresponding ATP hydrolysis [86]. For the active transport mechanism, the ATP has a cytosolic origin in ILC915, while in PpaMBEL1254 and iPP668 the required ATP is peroxisomal. According to [87, 88], peroxisomal ATP is only required for medium chain fatty acid activation, therefore long and very long chain fatty acids transport should be dependent on cytosolic ATP.

Each cycle of β-oxidation is represented by 4 reactions. Nevertheless, for unsaturated fatty acid degradation, and due to its highly complex degradation steps which depend on the position of its double bounds [89, 90], lumped reactions up to the generation of acetyl-CoA were taken from iPP668. However, the desaturation reaction of C_18:3_ to C_18:2_ was taken from iLC915.

### Sphingolipid metabolism

General sphingolipid biosynthetic pathways in yeast are partially homologous to *S*. *cerevisiae* and they are extensively described in the literature [91, 92]. Nevertheless, unlike *S*. *cerevisiae*, some yeasts such as *P*. *pastoris*, are able to produce glucosylceramides (GlcCer) from sphingolipid bases [93–96].

None of the three models include GlcCer biosynthesis. Ternes and co-workers [97, 98] identified the gene role in GlcCer pathway and described the fatty acid specific composition in GlcCer and other sphingolipids, as well as the main chain sphingoid bases in *P*. *pastoris*.

Sphingoid bases can be derived from palmitoyl-CoA and stearoyl-CoA. However, only a 5% of the detected species correspond to the last one. In fact, Ternes and co-workers [98] characterized sphingolipid composition assuming all the species were formed with a palmitoyl-CoA derived sphingoid base. This sphingolipid composition is in agreement with other literature sources [62, 99, 100]. As palmitoyl-CoA bases represents around 95% of sphingoid bases, only palmitoyl-CoA derived ones are taken into account in this model.

### Glycosylation pathways

Protein glycosylation pathways are not accurately described in previous models of *P*. *pastoris*. Only iLC915 partially included *N*-glycosylation, *O*-glycosylation and glycosylphosphatidylinositol-anchor (GPI-anchor) biosynthesis pathways. However, compartmentalization of several reactions of this pathway also required revision.

The first part of the *N*-glycosylation process is highly conserved among eukaryotes [101, 102]. It takes place in the cytosol up to the addition of 5 mannose residues (Man) forming (Man)5(GlcNAc)2(PP-Dol)1 oligosaccharide. At this point, the oligosaccharide is transferred to the endoplasmic reticulum (ER), where up to 9 Man and 3 glucose residues (Glc) are further added [103]. In the second and less conserved part of the pathway, the dolichol diphosphate attachment to the protein is represented by a pseudo-reaction forming the compound (Glc)3(Man)9(GlcNAc)2(Asn)1 in which Asn represents an asparagine residue from the targeted protein. Once the oligosaccharide is attached to the Asn residue of the target protein, it is further modified by the removal of one Man. The resulting glycoprotein is transported to the Golgi Apparatus [104, 105]. There, an heterogeneous pattern of glycosylation has been observed corresponding to the different heterologous proteins expressed in *P*. *pastoris* [19, 106, 107]. As an example, differences in Man residues range from 6 to 18 [108–110] and even may include hypermanosylation [111]. Due to its complexity and variability, in this model an average glycan is assumed to consist of (Man)9(GlcNAc)2(Asn). The resulting oligosaccharide contributes to the formation of a mannan (Man polymer represented by 1 mannose residue polymer) and a chitin (*N*-Acetylglucosamine polymer). Both contribute to the biomass formation as a specific component of the carbohydrate fraction.

Similarly to *N*-glycosylation in mannan formation, *O*-glycosylation is included assuming an average of 3 Man oligosaccharides [112–115]. *O*-glycosylation is also represented by a pseudo-reaction forming the compound (Man)1(Ser/Thr)1 in which Ser/Thr represents a serine or threonine residue of a protein.

Finally, GPI-anchor biosynthesis was also reviewed and compartments reassigned according to Orlean and Menon [116].

### Oxidative phosphorylation

There are *P*. *pastoris* specific traits in respiratory chain that should be included in the GEM. As an example, while *complex I* is not present in *S*. *cerevisiae* [117], it is described in *P*. *pastoris* [118, 119]. None of the previous *P*. *pastoris* models include respiratory *complex I* and so, an important proton translocation step was missing. In general, two main traits of oxidative phosphorylation were deeply analyzed: the proton stoichiometry and the complexes integrating the electron transport chain.

In this model the mitochondrial intermembrane space has been included. Hence proton translocation is assumed to occur from the mitochondrial matrix to the intermembrane space. Thus, protons pumped out from the mitochondria do not merge with the high amount of protons from the cytosolic space. Regarding the electron transfer chain reactions, each of the previous *P*. *pastoris* models shows a different stoichiometry for proton translocation. After a review of the stoichiometry and relevant literature it was decided to apply a stoichiometry that satisfies the H^+^ balance of the metabolites’ charged formula and including *complex I* stoichiometry considerations proposed by Wikström and Hummer [120]. This includes the *complex I* translocating 4 H^+^ to the intermembrane space [120, 121].

In the present model, reactions for non H^+^ translocating outer and inner mitochondrial membrane NAD(P)H dehydrogenases (cytoplasmic side and matrix side) were also included. While inner NADH dehydrogenase appears in all three models, outer NADH dehydrogenase was only present in iPP668 and PpaMBEL1254, despite both dehydrogenases have been previously reported [118, 122, 123]. We also included PAS_chr1-4_0299 putative NADPH dehydrogenase homologue to *Kluyveromyces lactis* [124, 125] and also included in its metabolic reconstruction [126].

On the other hand, *complex III* and *IV* are included in all three models, but several discrepancies exist on the details of the H^+^ balance due to the consideration of alternative metabolite’s molecular formulas or the result of using different criteria when considering chemical and translocated protons [127]. An additional trait for *complex III* equations is the complexity on the stoichiometric representation of Q-cycle [128, 129]. Therefore, in this model stoichiometric coefficients of equations for *complex III* and *IV* reactions were chosen with special attention to the proton balance. The selected stoichiometry was of 2H^+^/2e^-^ for *complex III* and 4H^+^/2e^-^ for *complex IV* according to recent literature [121, 128, 129].

One additional characteristic of *P*. *pastoris* is the presence of an alternative oxidase that could bypass *complex III* and *IV* in the respiratory chain [117, 130] which seems to be active only in certain growth conditions [117, 122, 131]. Although this oxidase was only included iLC915, our consensus model also incorporates this reaction in the electron transport chain module. Finally, the stoichiometry for ATP synthase was maintained as in PpaMBEL1254 and iPP668 (4H^+^/ATP, resulting in a final maximum theoretical stoichiometry of 2.5e^-^/ATP) as its H^+^ balance is in agreement with the available literature [132].

### Other reviewed pathways

Metabolization of some sugars was also updated. Sugars such as starch, maltose or cellobiose were able to be assimilated in some of the previous models. However, Kurtzman [44] and Naumov and co-workers [133] characterized substrate assimilation in *P*. *pastoris* and reported non growth for these carbon sources. Consequently reasons for their metabolization were revised and discarded reactions are detailed in S4 File. L-rhamnose assimilation was only possible in PpaMBEL1254. However the specific metabolic steps included for its metabolization were not typical of yeast species. Therefore alternative reactions and genes associated to the metabolization of L-rhamnose were added as suggested in [134], also described similarly for *P*. *stipitis* [135, 136].

A full list of modifications from the original models, also including some additional pathways and subsystems not discussed above such as phosphatidylinositol synthesis or transport reactions, is provided in S4 File.

### General characteristics of the model

After following all the steps described above, an extended model (iMT1026) is obtained that integrates previous *P*. *pastoris’* GEMs, which is provided in S1 and S5 Files and is also available at BIOMODELS database (MODEL1508040001) [58]. The general characteristics of the resulting model are described in the following.

In the first place this model includes an increased number of gene-protein-reaction relationships as can be seen in Table 1.

**Table 1:** Comparison of the main features of iMT1026 and previous *P*. *pastoris’* GEMs.

	iPP668	PpaMBEL1254	iLC915	iMT1026
**Genes**	668	540	915	1026
**Metabolites** ^a^	1177 (684)	1058 (696)	1302 (899)	1689 (1018)
**Reactions**	1354	1254	1423	2035
Cytosolic	623	604	790	1059
Mitochondrial	163	155	205	268
Peroxisomal	66	66	64	102
Extracellular	12	11	0	16
Endoplasmic reticulum	15	7	34	41
Golgi apparatus	4	8	4	13
Vacuolar	3	6	12	9
Nuclear	16	17	0	17
Intercompartmental/Transport	452	328	314	510

^a^ Total number of metabolites, with compartment, and unique metabolites (in brackets).

**Fig 2:**
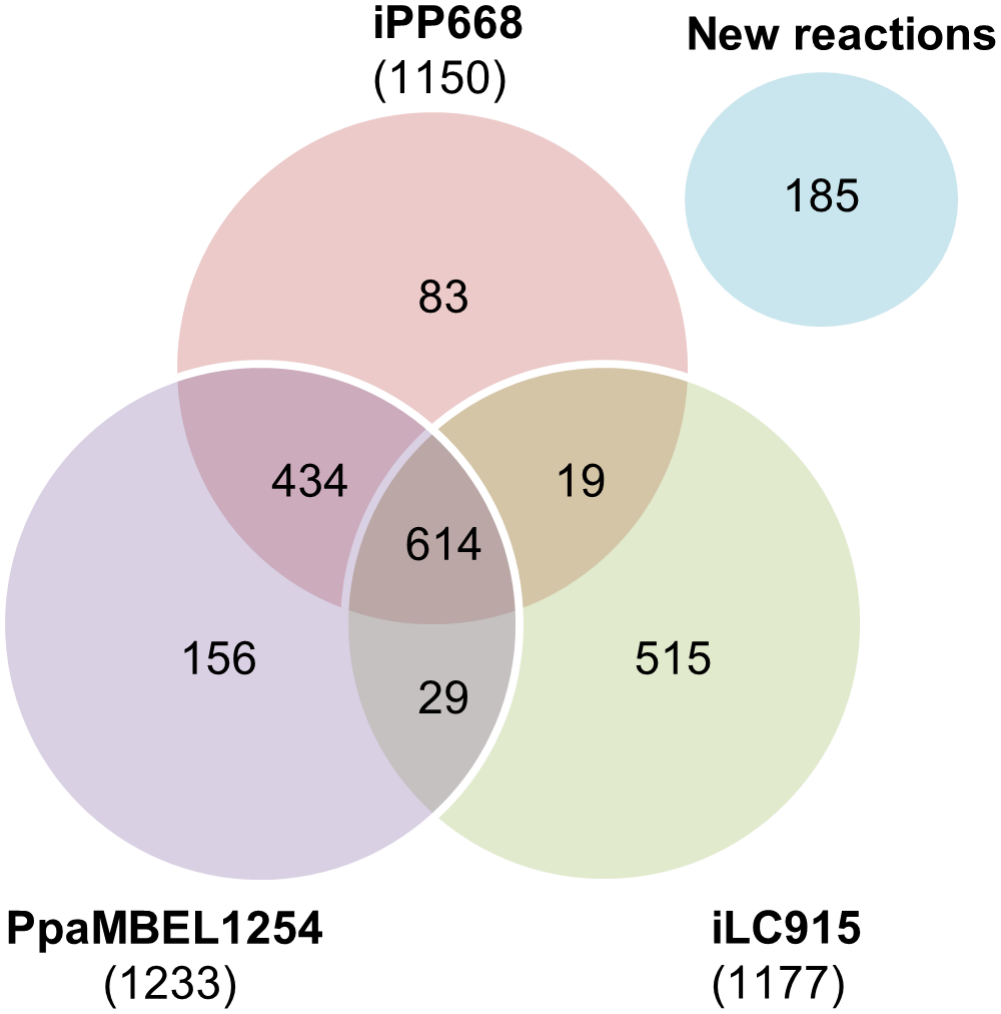
Reactions from PpaMBEL1254, iPP668 and iLC915 included in iMT1026 model.

Our final model incorporated 185 new reactions that didn’t appear in previous models and has 614 common reactions in all three models (Fig 2). Reactions appearing in Fig 2 as common to two or all three different models include those reactions that have been taken directly from the previous model. Therefore, they do not include those reactions that are either not the same but equivalent, have been decomposed in several reactions or are a combination of several other reactions. Thus, taking into account these multi-step, lumped or decomposed reactions, in the final model there are up to 721 equivalent reactions in common in all three models, 504 in two of the three models and 638 reactions in only one of the models, without any clear equivalence in any of the other two models.

### Maintenance and growth-associated ATP calculations

As described in Materials and Methods, data from [24, 60, 69, 70] was used to calculate the NGAME. As an initial step, a value of 32 mol ATP/ mol glucose, was obtained by maximizing the ATP turnover using 1 mmol/(gDCW·h) of glucose. The *y*-intercept from a representation of glucose uptake rate versus growth rate was 0.0878 mmol glucose/(gDCW·h) which corresponds to 2.81 mmol ATP/(gDCW·h) using the glucose-ATP conversion factor obtained in the initial step. This calculated glucose NGAME value is similar to the 2.3 mmol ATP/(gDCW·h), previously proposed by [31] for glucose, and close to the NGAME estimated for *Pichia* (*Scheffersomyces*) *stipitis* also growing in glucose [137].

On the other hand, GAME for glucose was estimated by fitting the calculated biomass-substrate yields to experimental data. This way a value of 72 mmol ATP/gDCW was obtained. This amount of ATP associated to cell growth is also similar to 70.5 mmol ATP/gDCW calculated previously for *P*. *pastoris* [33] and close to the experimentally calculated values for *S*. *cerevisiae* of 62–71 mmol ATP/gDCW [138, 139], and to the 69.2 mmol ATP/gDCW, *in silico* estimated [5].

For the case of the glycerol and methanol co-feeding cultivations, ATP maintenance values calculated by Jordà and co-workers [61] were used as NGAME as available data was insufficient for a new determination. These values range from 4.5 to 5.7 mmol ATP/gDCW and are similar to 6 mmol ATP/gDCW, proposed in iLC915 [33]. For the different conditions tested, specific GAMEs were calculated by fitting the simulations to experimental data from [61]. These experimental data show that the ratio of the glycerol or methanol uptake rates with the growth rate is different for each pair of glycerol:methanol feeding conditions (80:20, 60:40 and 40:60, % w/w at 0.05 and 0.16 h^-1^ growth rates), therefore a specific GAME was calculated for each case. The obtained values ranged within the 69.8 and 125.6 mmol ATP/gDCW interval. These GAME values increase with the fraction of methanol in the mixed feeding and are in agreement with those calculated by Caspeta and co-workers [33], who estimated a maximum GAME for methanol as sole carbon source of 150 mmol ATP/gDCW.

### Carbon source assimilation

The model agreement with *P*. *pastoris* utilization of different carbon sources was tested and compared to experimental data [44, 133]. A total of 47 carbon sources were evaluated (Table 2) using an *in silico* minimal medium, with ammonium, phosphate and biotin. The model successfully predicts carbon assimilation for all sources tested.

**Table 2:** Evaluation of the substrate assimilation capabilities in *P*. *pastoris*.

	Experimental ^a^	*In silico*
D-glucose	+	+
DL-lactate	+	+
Mannitol	+	+
Ethanol	+	+
Glycerol	+	+
L-Rhamnose	+	+
Methanol	+	+
Succinate	+	+
Trehalose	+	+
Sorbitol	+	+
Citrate	v	+
D-Xylose	- / + ^b^	+
Xylitol	- / + ^c^	+
5-keto-D-gluconate	-	-
Arbutin	-	-
Cellobiose	-	-
D-arabinose	-	-
D-Galacturonate	-	-
D-gluconate	-	-
D-glucono-1,5-lactone	-	-
D-Glucosamine	-	-
D-Glucuronate	-	-
D-Ribose	-	-
Erythrol	-	-
Galactitol	-	-
Galactose	-	-
Hexadecane	-	-
*myo*-Inositol	-	-
inulin	-	-
Lactose	-	-
L-Arabitol	-	-
L-Arabinose	-	-
L-Sorbose	-	-
L-Tryptophan	-	-
Maltose	-	-
Meleziose	-	-
Melibiose	-	-
methyl-alpha-D-glucoside	-	-
N-acetylglucosamine	-	-
Raffinose	-	-
Ribitol	-	-
Saccharate	-	-
Salicin	-	-
Soluble starch	-	-
Sucrose	-	-

+, carbon assimilation and growth; -, no carbon assimilation; v, variable growth/non-growth experiments observed.

^a^ Experimental data from [44, 140].

^b^ Described in [141].

^c^ Described in [142].

### Reaction essentiality

**Fig 3:**
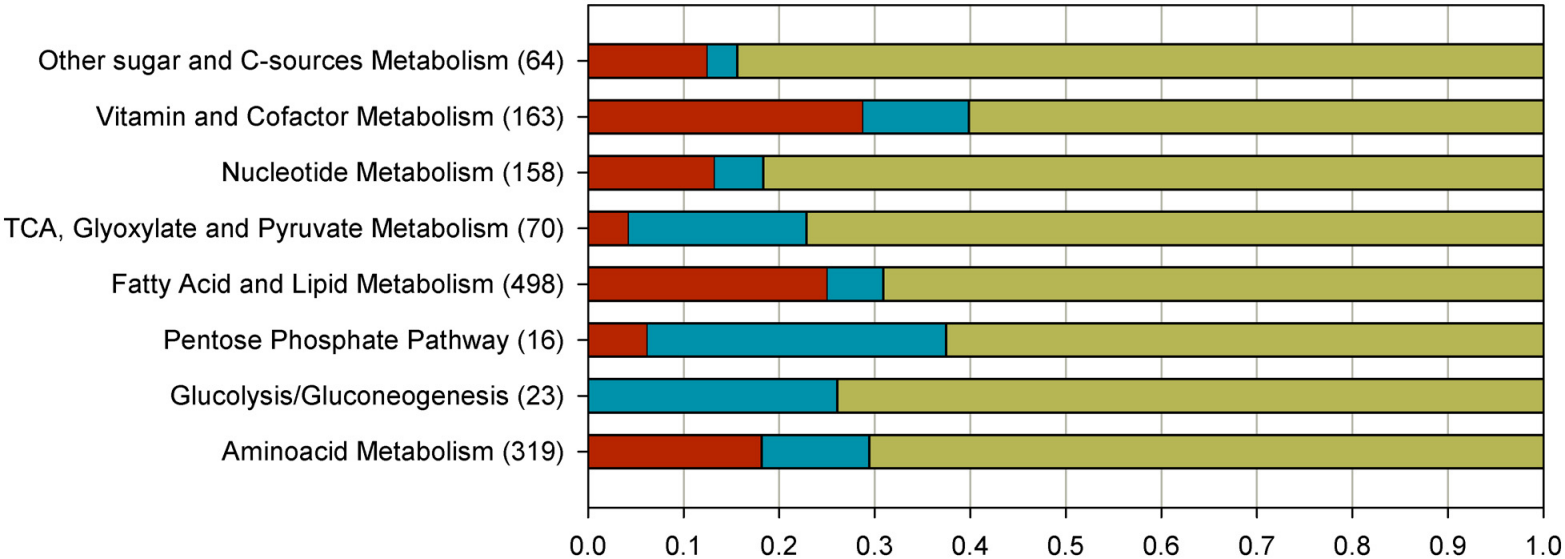
Summary of reaction essentiality results for glucose simulations grouped into major pathways. FBA was performed by optimizing biomass production and sequentially constraining to 0 each reaction in the corresponding simulations. The resulting growth rate was compared with the wild type one. Metabolic reactions were classified in three categories according to the relative growth rate obtained: Essential (E), partially-essential (PE) and non-essential (NE). X axis represent the fraction of each type of reactions in each category of E (in red), PE (in blue) and NE (in green). Reactions are distributed in 8 major subsystems (Y axis). Numbers between brackets indicate number of reactions in each group. Equivalent figures for oxygen limiting conditions and glycerol:methanol simulations can be found in S1 Fig.

An interesting trait of the obtained GEMs is the identification of those reactions critical or essential for biomass growth (essential reactions). Simulations to determine reaction essentiality were performed for glucose in normoxia, limited oxygen and hypoxia conditions and also for mixtures of glycerol and methanol at different growth rates. No significant difference in reaction essentiality was observed in all the conditions tested. Thus, similar patterns of distribution of essential reactions in each pathway are observed for all the cases. The results, grouped into major metabolic pathways, are summarized in Fig 3 (and S1 Fig). From a global point of view, essential reactions represent a 15–16% of the total reactions, while 76–79% of the reactions are classified as non-essential. The remaining 6–9% are partially-essential and its deletion causes a decrease in growth rate.

The results show that there are 312 condition independent essential reactions, which are essential in all the performed simulations. These essential reactions can be grouped in three main groups: in the first group, most of them are associated with lipid metabolism (40%), in the second group most reactions belong to the amino acid metabolism (18.6%) while the third group mostly includes cofactor related essential reactions as a 14.1% of all common essential reactions. Similarly to other GEM models, such as *S*. *cerevisiae*, extending the model and including more detailed biomass composition results in an increase of essential reactions directly linked to the biomass related metabolites. Nevertheless, this essentiality could be overestimated *in silico*, due to the fact that *in vivo* systems are able to replace de missing species with other similar biomass components.

### Model validation

An additional step of model validation was performed by comparing the model predicted values with an additional set of experimental data including diverse combinations of glucose, glycerol and methanol chemostats [61, 66].

**Fig 4:**
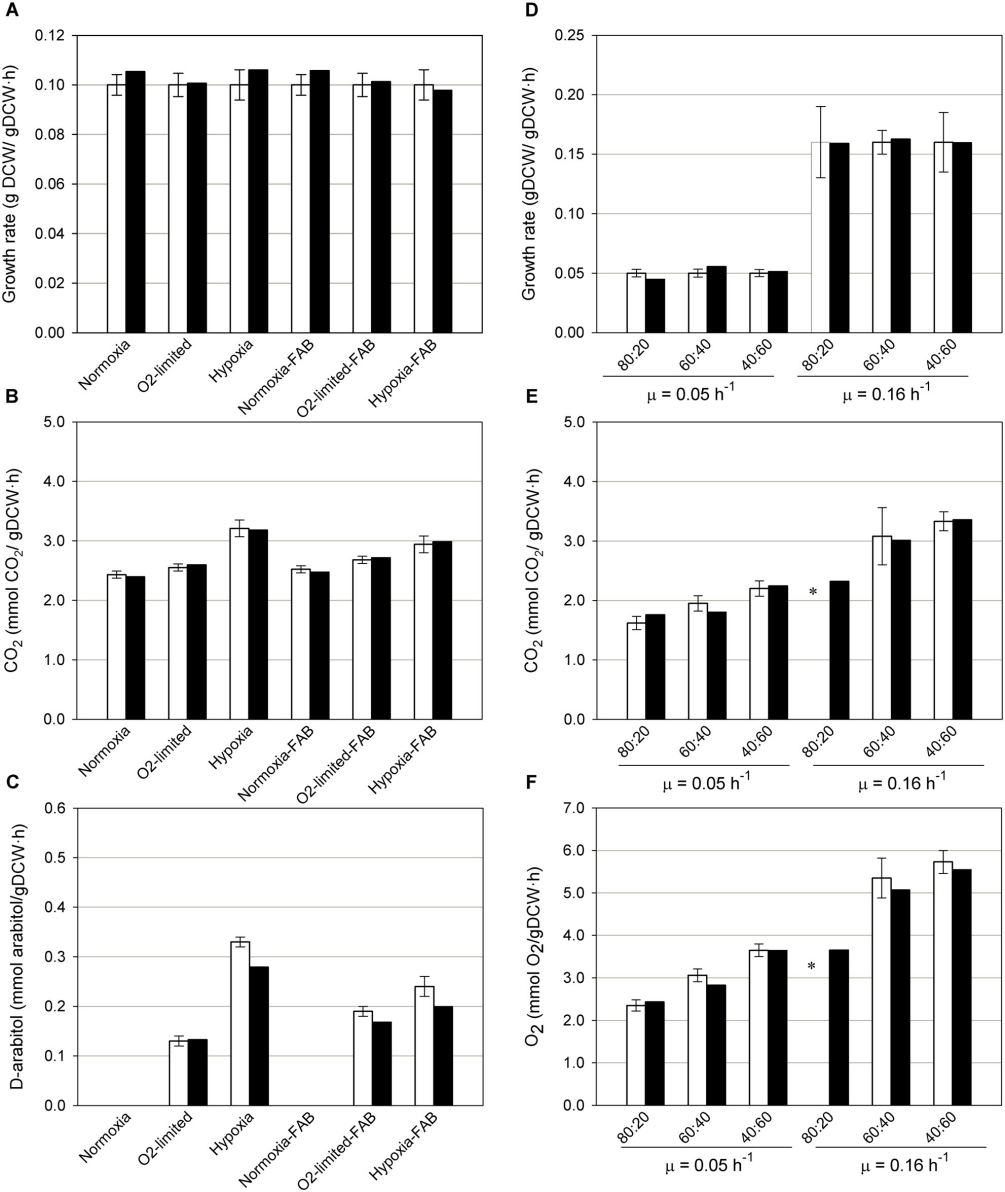
Results of the model validation. Graphs with (A) growth rate (B) CO_2_ and (C) D-arabitol production predictions when simulating glucose chemostats at different oxygen conditions, with and without recombinant protein production [60, 66] with glucose, O_2_ and ethanol fluxes constrained to the experimental values. (D) Growth rate (E) CO_2_ production and (F) O_2_ consumption predictions when simulating different glycerol:methanol chemostats [25, 61]. White and black bars correspond to experimental and predicted data respectively.* Not determined in [25].

For the glucose chemostats, simulations with different oxygen availabilities, growth rates and CO_2_ production were successfully predicted with errors lower than 6% for both FAB expressing and non-expressing strains (Fig 4A, 4B and 4C). According to the available experimental data [60, 66], when the oxygen availability decreases ethanol and other metabolites are secreted. The present model is also able to predict by-product formation when oxygen-limited conditions are simulated. However, the model predicts a slightly higher production of ethanol and none of the other by-products, such as arabitol or pyruvate. Nevertheless, when ethanol secretion flux is constrained to the experimentally measured value, arabitol secretion is also predicted (Fig 4C). In addition to arabitol, pyruvate is also secreted in the *in silico* predictions when both ethanol and arabitol are constrained to the experimental values. This discrepancy of the model to directly predict arabitol or pyruvate secretion if no additional constraint is imposed points to additional regulatory constraints other than those strictly stoichiometric. In addition, different cofactor utilization by combinations of isoenzymes [143, 144], and their impact on NAD(P)^+^/NAD(P)H regeneration, could be one of the key factors for the production of those alternative products [145, 146]. Constraining the model with additional ^13^C-labelling data [147] as well as studying the impact of cofactor perturbation and analyzing these cofactor demands for cell growth, as done in other organisms [148] would be interesting approaches to consider in future studies.

For the second dataset, (glycerol:methanol mixtures), specific growth rate, together with specific O_2_ consumption and CO_2_ production rates were predicted within a 11% of deviation, as shown Fig 4E, 4F and 4G. Similarly to the above described glucose tests, arabitol was only produced when ethanol was constrained to the experimental values; otherwise, ethanol is the preferred product of the stoichiometric model. As in the previous glucose case these results point to another possible level of regulation for arabitol production not included in the model, as without it ethanol production appears as the most efficient way to regenerate NAD^+^ for maximum biomass production.

In order to compare the accuracy of our model with the previous existing models a set of simulations were performed for glucose and glycerol:methanol cultivations (S5 File). The same constrains were set to all the models and growth rate, CO_2_ production and O_2_ consumption (only in the glycerol:methanol simulations) were compared to the experimental values [61, 66]. As shown in S5 File, iMT1026 can predict the evaluated macroscopic cultivation parameters more accurately, i.e. with smaller deviations from experimental data. Moreover, our model is also able to describe byproduct secretion under respirofermentative conditions.

## Conclusions

In summary, a consensus GEM of the yeast *P*. *pastoris* integrating the three preexisting models has been obtained. Importantly, the new GEM, iMT1026, is more complete and includes a comprehensive revision and upgrading of several metabolic processes (e.g. fatty acid and sphingolipid metabolism, protein glycosylation and energy metabolism) based on new information emerged from recent literature. Furthermore, the new GEM has been validated using different sets of experimental data corresponding to a wider range of physiological states than previous GEMs. From our point of view this GEM improves the capabilities in terms of accuracy of predictions/simulations in relation to previous models. Overall, we provide an improved tool to the *P*. *pastoris* community for the physiological analysis and understanding of this yeast. It is expected that on-going efforts in the functional (re)annotation of the *P*. *pastoris* genome will allow for further improvements of its GEMs by all the *P*. *pastoris* community. From a wider perspective, it also has to be pointed out the importance of curating and manually revising new GEMs of non-model organisms that are based on GEM scaffolds from related model organisms. Despite the comprehensiveness of these scaffolds, an exhaustive analysis of specific metabolic traits of the non-model organism is still essential to construct a GEM describes/predicts its metabolic phenotype accurately.

## Supporting Information

S1 Fig Reaction essentiality analysis. Summary of reaction essentiality results grouped into major pathways.
(PDF)

S1 File iMT1026 model. *Pichia pastoris* GEM model in xlsx format for use in the RAVEN toolbox.
(XLSX)

S2 File Biomass composition. Details of biomass composition for cultivations on glucose and glycerol:methanol mixtures.
(XLSX)

S3 File Recombinant protein composition. DNA, RNA and amino acid compositions for the FAB and ROL recombinant proteins expressed in *P*. *pastoris* and tested in the model.
(XLSX)

S4 File Reaction changes. List of modified, added and excluded reactions in iMT1026.
(XLSX)

S5 File iMT1026 model in SBML format. *Pichia pastoris* GEM model in SBML format generated with the RAVEN toolbox.
(XML)

S6 File GEMs performance comparison. Analysis of the prediction capabilities and deviations from experimental data in simulations performed with iMT1026 and the previous models.
(PDF)
