# Supplementary material for: Integration and Validation of the Genome-Scale Metabolic Models of Pichia pastoris: A Comprehensive Update of Protein Glycosylation Pathways, Lipid and Energy Metabolism
Source: PLoS One. 2016 Jan 26;11(1):e0148031. doi: 10.1371/journal.pone.0148031 (PMC4734642; doi:10.1371/journal.pone.0148031)
Supplement: S1 Fig — Summary of reaction essentiality results grouped into major pathways. (PDF) [file pone.0148031.s001.pdf]

S1 Fig. Reaction essentiality analysis in different cultivation conditions.

FBA was performed optimizing biomass production and sequentially constraining to 0 each reaction in the corresponding simulations. The resulting growth rate was compared with the wild type growth and reactions were classified in three categories according to the relative growth rate obtained: essential (E), partially essential (PE) and non-essential (NE). Reactions are grouped in 8 major pathways or global subsystems (Y axis). In brackets, the number of reactions included in each subsystem. X axis represent the fraction of each type of reactions in each category of E (in red), PE (in blue) and NE (in green). Reaction essentiality was evaluated with glucose chemostats with different oxygen conditions and glycerol:methanol mixtures chemostats: (A) Glucose and normoxia; (B) Glucose and limited oxygen; (C) Glucose and hypoxia; (D) Glycerol:methanol (80:20 w/w) at μ=0.05 h^{-1}; (E) Glycerol:methanol (60:40 w/w) at μ=0.05 h^{-1}; (F) Glycerol:methanol (40:60 w/w) at μ=0.05 h^{-1}; (G) Glycerol:methanol (80:20 w/w) at μ=0.16 h^{-1}; (H) Glycerol:methanol (60:40 w/w) at μ=0.05 h^{-1}; (I) Glycerol:methanol (40:60 w/w) at μ=0.05 h^{-1}.

A

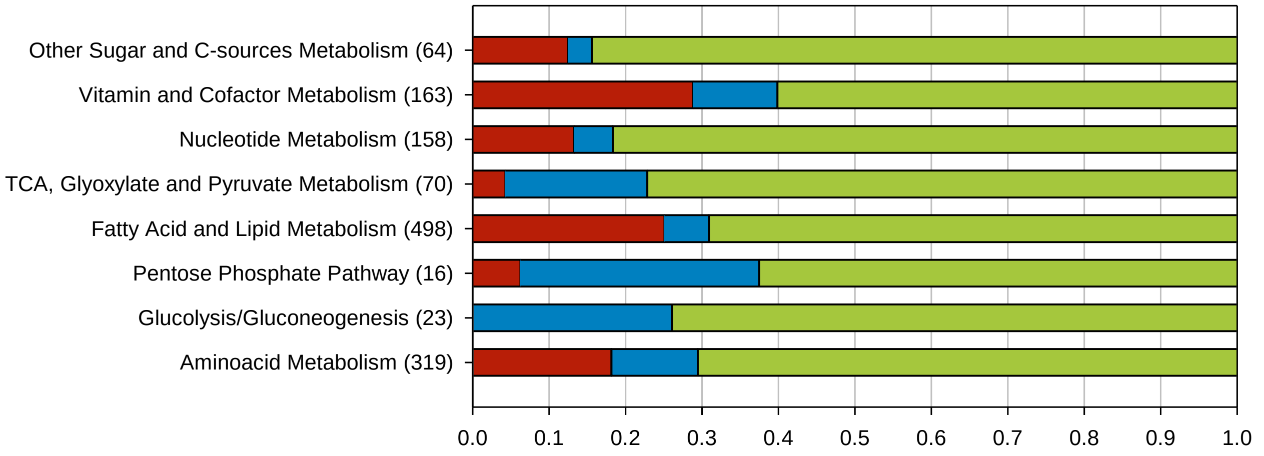

B

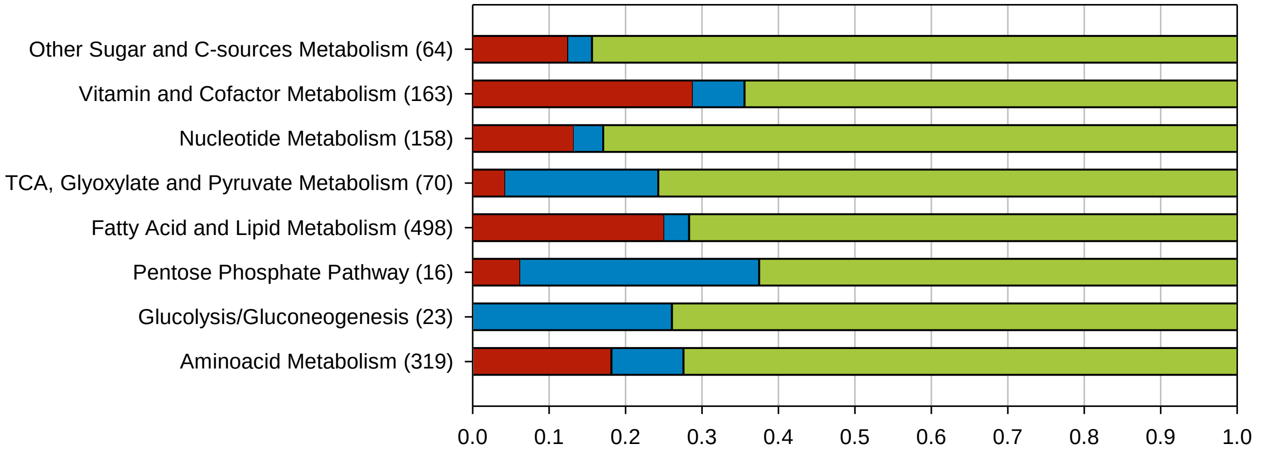

C

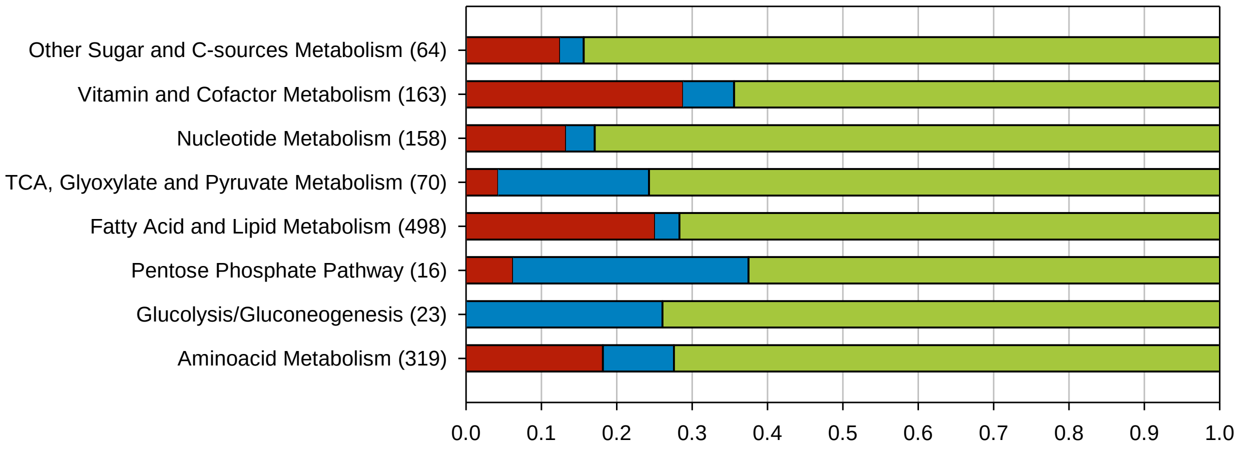
Other Sugar and C-sources Metabolism (64)
Vitamin and Cofactor Metabolism (163)
Nucleotide Metabolism (158)
TCA, Glyoxylate and Pyruvate Metabolism (70)
Fatty Acid and Lipid Metabolism (498)
Pentose Phosphate Pathway (16)
Glucolysis/Gluconeogenesis (23)
Aminoacid Metabolism (319)
0.0 0.1 0.2 0.3 0.4 0.5 0.6 0.7 0.8 0.9 1.0

D

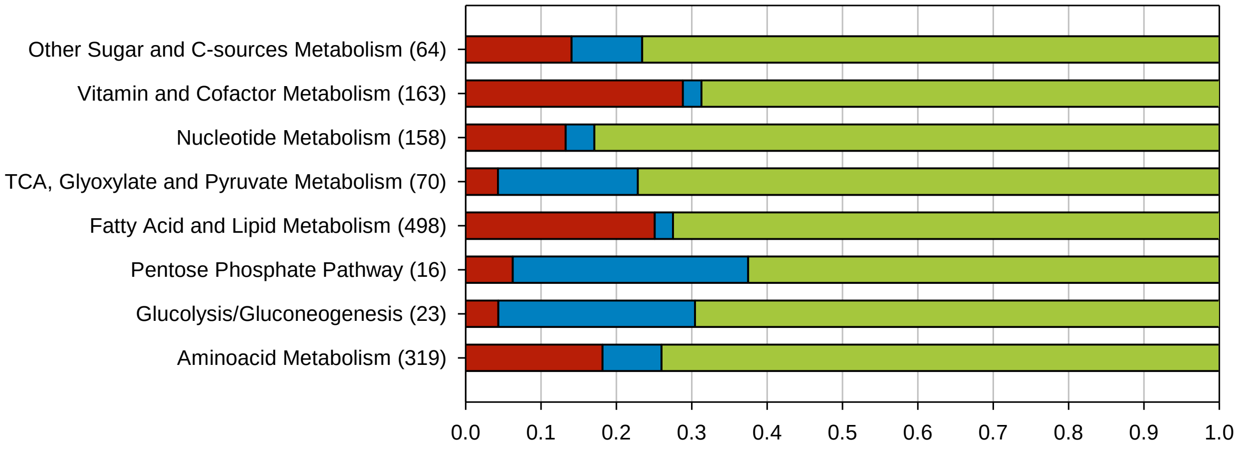
Other Sugar and C-sources Metabolism (64)
Vitamin and Cofactor Metabolism (163)
Nucleotide Metabolism (158)
TCA, Glyoxylate and Pyruvate Metabolism (70)
Fatty Acid and Lipid Metabolism (498)
Pentose Phosphate Pathway (16)
Glucolysis/Gluconeogenesis (23)
Aminoacid Metabolism (319)
0.0 0.1 0.2 0.3 0.4 0.5 0.6 0.7 0.8 0.9 1.0

E

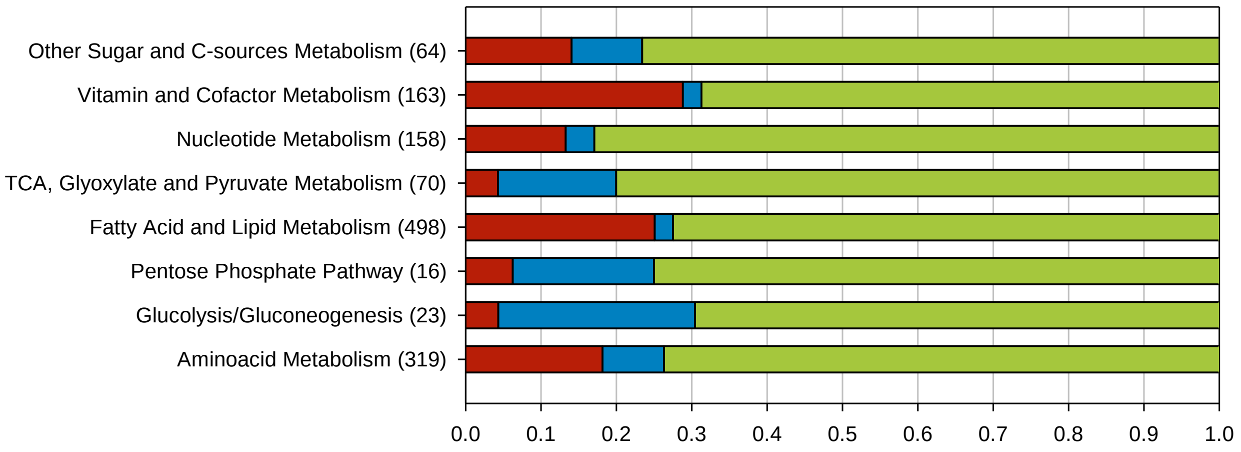
Other Sugar and C-sources Metabolism (64)
Vitamin and Cofactor Metabolism (163)
Nucleotide Metabolism (158)
TCA, Glyoxylate and Pyruvate Metabolism (70)
Fatty Acid and Lipid Metabolism (498)
Pentose Phosphate Pathway (16)
Glucolysis/Gluconeogenesis (23)
Aminoacid Metabolism (319)
0.0 0.1 0.2 0.3 0.4 0.5 0.6 0.7 0.8 0.9 1.0

F

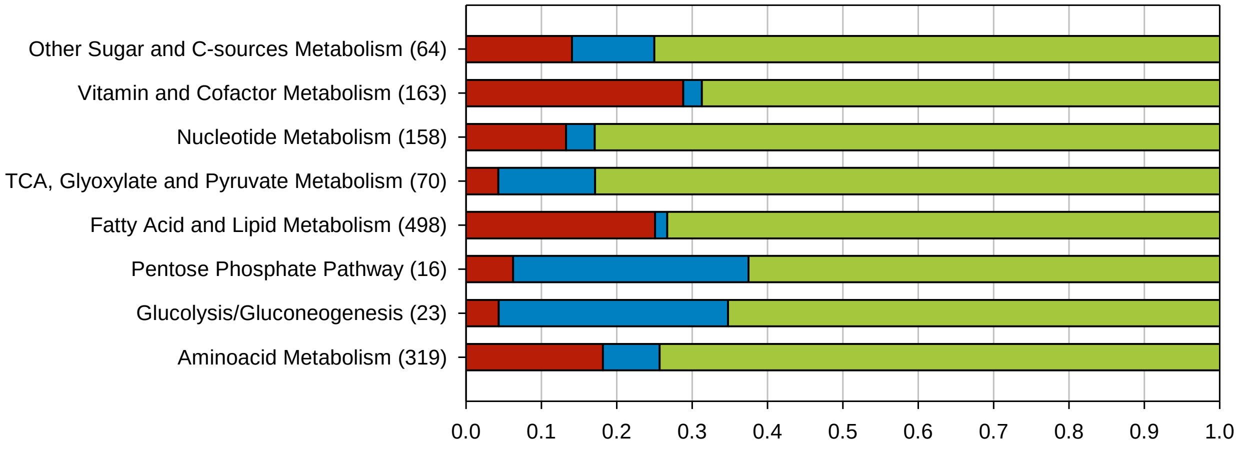
Other Sugar and C-sources Metabolism (64)
Vitamin and Cofactor Metabolism (163)
Nucleotide Metabolism (158)
TCA, Glyoxylate and Pyruvate Metabolism (70)
Fatty Acid and Lipid Metabolism (498)
Pentose Phosphate Pathway (16)
Glucolysis/Gluconeogenesis (23)
Aminoacid Metabolism (319)
0.0 0.1 0.2 0.3 0.4 0.5 0.6 0.7 0.8 0.9 1.0

G

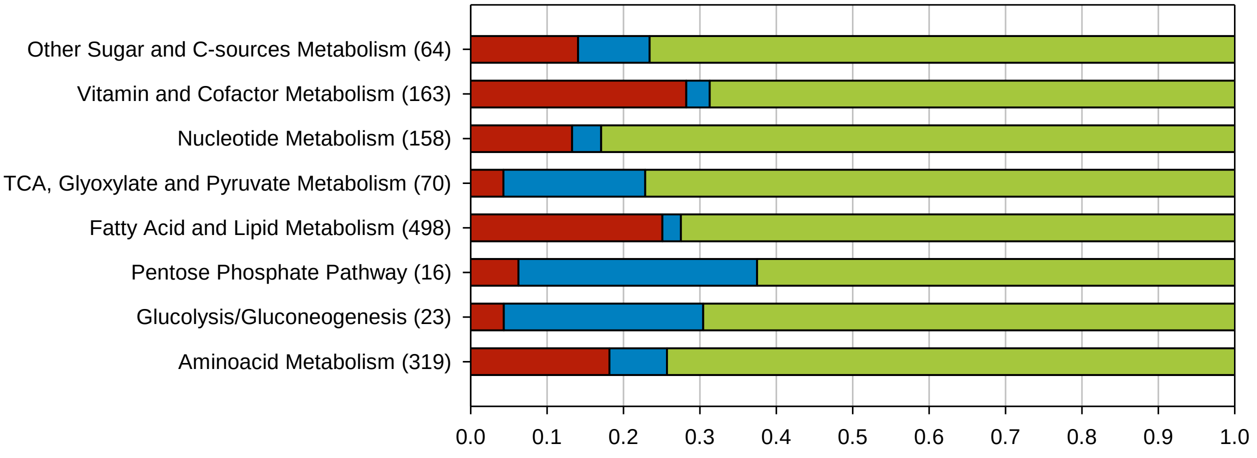
Other Sugar and C-sources Metabolism (64)
Vitamin and Cofactor Metabolism (163)
Nucleotide Metabolism (158)
TCA, Glyoxylate and Pyruvate Metabolism (70)
Fatty Acid and Lipid Metabolism (498)
Pentose Phosphate Pathway (16)
Glucolysis/Gluconeogenesis (23)
Aminoacid Metabolism (319)
0.0 0.1 0.2 0.3 0.4 0.5 0.6 0.7 0.8 0.9 1.0

H

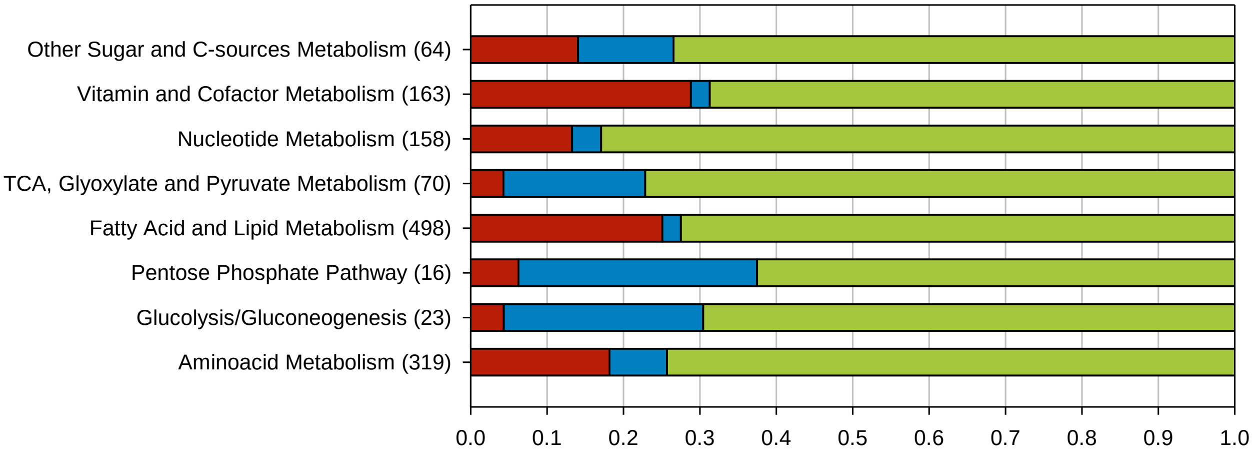
Other Sugar and C-sources Metabolism (64)
Vitamin and Cofactor Metabolism (163)
Nucleotide Metabolism (158)
TCA, Glyoxylate and Pyruvate Metabolism (70)
Fatty Acid and Lipid Metabolism (498)
Pentose Phosphate Pathway (16)
Glucolysis/Gluconeogenesis (23)
Aminoacid Metabolism (319)
0.0 0.1 0.2 0.3 0.4 0.5 0.6 0.7 0.8 0.9 1.0

I

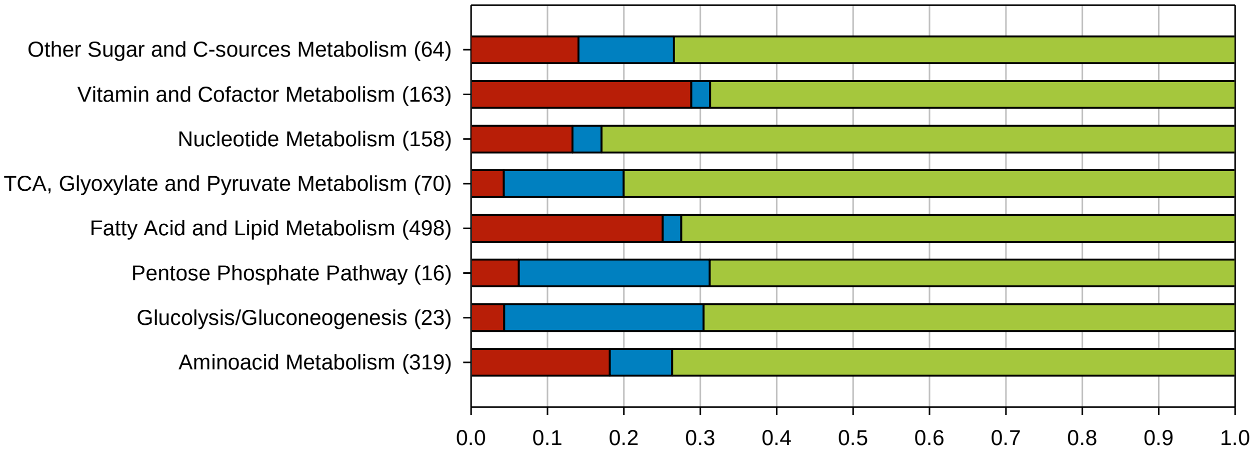
Other Sugar and C-sources Metabolism (64)
Vitamin and Cofactor Metabolism (163)
Nucleotide Metabolism (158)
TCA, Glyoxylate and Pyruvate Metabolism (70)
Fatty Acid and Lipid Metabolism (498)
Pentose Phosphate Pathway (16)
Glucolysis/Gluconeogenesis (23)
Aminoacid Metabolism (319)
0.0 0.1 0.2 0.3 0.4 0.5 0.6 0.7 0.8 0.9 1.0
