# Supplementary material for: Integration and Validation of the Genome-Scale Metabolic Models of Pichia pastoris: A Comprehensive Update of Protein Glycosylation Pathways, Lipid and Energy Metabolism
Source: PLoS One. 2016 Jan 26;11(1):e0148031. doi: 10.1371/journal.pone.0148031 (PMC4734642; doi:10.1371/journal.pone.0148031)
Supplement: S6 File — Analysis of the prediction capabilities and deviations from experimental data in simulations performed with iMT1026 and the previous models. (PDF) [file pone.0148031.s007.pdf]

Supporting information S6 File

GEMs performance comparison

Models were compared by flux balance analysis performing a maximization of biomass production (x) under the constraints of the experimental variables as described below.

Simulations parameters

Glucose – O_2 levels

Glucose and oxygen uptake rates were constrained according to [1]. Ethanol production was also constrained for O_2-limited and hypoxic cases. Only the wild type (wt) strain was evaluated. Biomass growth and CO_2 production were calculated by the model.

Glycerol:methanol mixtures

Only glycerol and methanol uptake rates were constrained for the glycerol and methanol cases according to [2]. When arabitol is experimentally produced, its measured production rate is set as lower bound for arabitol secretion only for models iMT1026 and iLC915. The other two models, iPP668 and PpaMBEL1254, are not able to generate arabitol.

Simulations results

Glucose – O_2 levels

By setting the normoxic conditions in iLC915, some flux loops occur which were avoided using the following strategy. Reactions involving bidirectional formation of DNA[m], DNA[c], RNA[c] and RNA[m] by a single nucleotide were set to 0. Otherwise reactions such as Diphosphate[c] + DNA[c] <=> dATP[c] could occur without energetic costs. Ethanol is also produced in these conditions.

First constrain set of constrains for iLC915 (instructions for COBRA Toolbox [3]):

iLC915b=changeRxnBounds(iLC915E,{'r66','r910','r1104','r239','r111','r106','r490','r791','r243','r252','r253','r307','r308','r404','r405','r1320','r639','r640','r641','r642','r649','r650','r651','r652','r645','r646','r643','r644','r653','r654','r655','r656','r534'},0,'b').

With this constrains iLC915 can predict biomass growth with glucose.

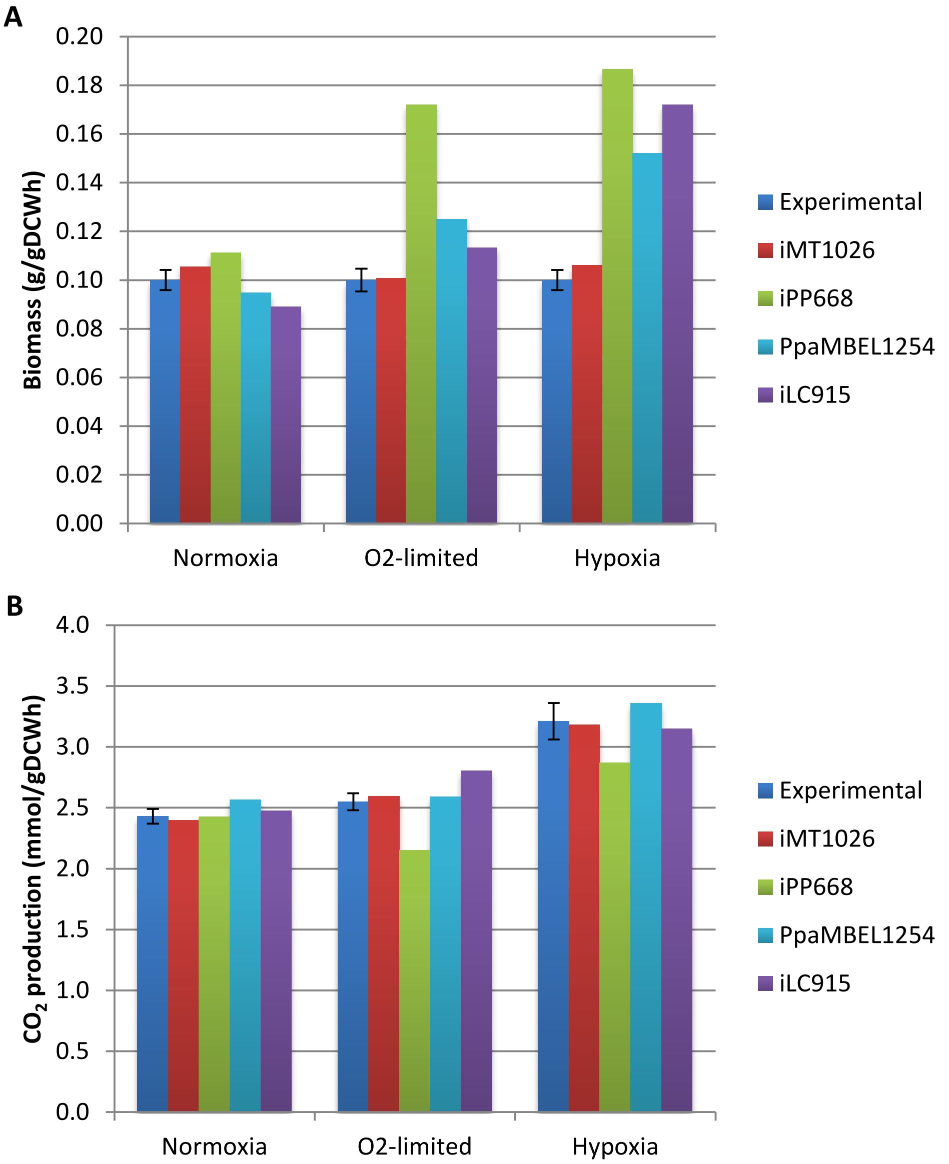

Fig S6.1. Comparison of predicted values for each model with experimental data. Graphs with (A) growth rate (B) CO_2 production predictions simulating glucose chemostats at different oxygen conditions with and without recombinant protein production with glucose, O_2 and ethanol fluxes constrained to the experimental values.

When simulating oxygen restricted conditions, none of the previous models predict arabitol secretion when ethanol production is constrained, and iMT1026 is the only one that predicts this by-product secretion.

Glycerol:methanol mixtures

To compare the performance of the models using mixtures of glycerol:methanol as carbon sources, the experimental data corresponding to the strain *P. pastoris X33 pGAPZαA_ROL* which expresses a *Rhizopus oryzae* lipase were used. Due to the fact that none of the other models incorporate ROL production equations, the simulation using iMT1026 with no ROL production was also performed. Comparing iMT1026 expressing ROL and iMT1026 without the ROL expression, similar values were observed in the evaluated fluxes. Thus, to compare the accuracy of the model using these experimental data, iMT1026 was used setting the expression of ROL to zero.

iLC915 has also to be constrained, for the above explained reasons, for the simulation of glycerol:methanol feeding conditions (instructions for COBRA Toolbox [3]):

iLC915=changeRxnBounds(iLC915E,{'r639','r640','r641','r642','r649','r650','r651','r652','r645','r646','r643','r644','r653','r654','r655','r656'},0,'b')

For these tests PpaMBEL1254 has to be modified. Reaction of O_2 diffusion to peroxisomes has to be added to enable growth and assimilation of methanol. Thus, results showed represent the modified PpaMBEL1254, otherwise no growth was reported.

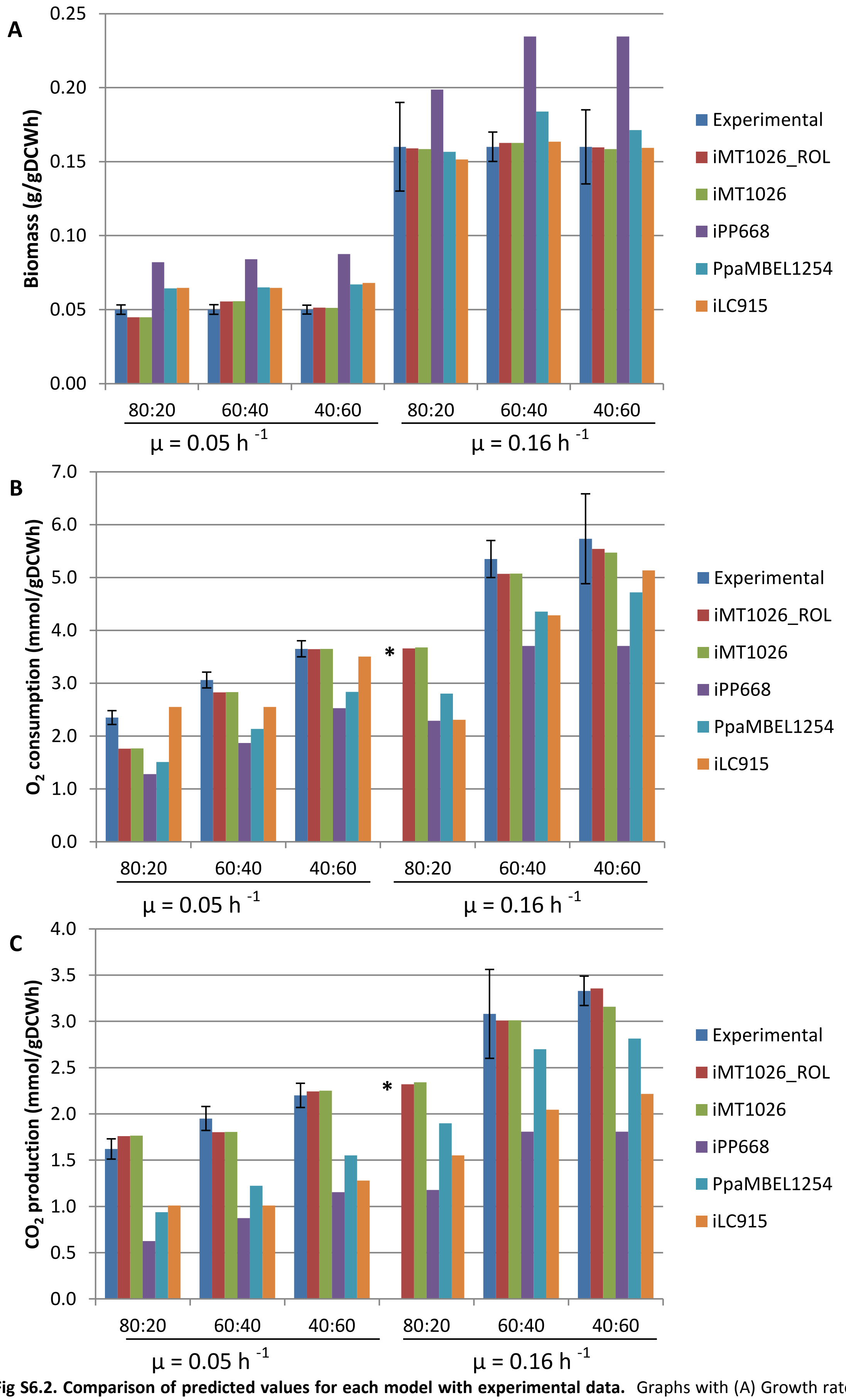

Fig S6.2. Comparison of predicted values for each model with experimental data. Graphs with (A) Growth rate (B) O_2 consumption and (C) CO_2 production predictions simulating different glycerol:methanol chemostats. * Not determined.

Model evaluation

For evaluating and comparing all the models, a statistical parameter (*eq.* 1) was calculated for all the predicted variables: biomass formation and CO_2 production in the glucose and different O_2 levels case; and biomass formation, O_2 consumption and CO_2 production in the glycerol:methanol simulations.

$$\frac{1}{n} \cdot \sum_{i=1}^{n} \frac{\sqrt{\left(v_{s_i} - v_{e_i}\right)^2}}{v_{e_i}} \% \qquad (eq\ 1)$$

Where v_{s_i} is the resulting flux from the simulation for the variable i and v_{e_i} is the experimental flux for this variable. The total number of predicted and compared fluxes with the experimental values is n and equals to 21.

Table S6.1. Comparison of deviations from experimental values. Calculations were performed according to (*eq.* 1)

	iMT1026_ROL	iMT1026	iPP668	PpaMBEL1254	iLC915
**Glucose**		2.71%	32.73%	15.66%	18.30%
**Glycerol:methanol**	6.90%	7.27%	48.93%	26.40%	25.14%
**Overall**		**6.08%**	**44.70%**	**23.60%**	**23.36%**

References

1. Carnicer M, ten Pierick A, van Dam J, Heijnen JJ, Albiol J, van Gulik W, et al. Quantitative metabolomics analysis of amino acid metabolism in recombinant Pichia pastoris under different oxygen availability conditions. Microb Cell Fact. 2012;11: 83. doi:10.1186/1475-2859-11-83

2. Jordà J, De Jesus SS, Peltier S, Ferrer P, Albiol J. Metabolic flux analysis of recombinant *Pichia pastoris* growing on different glycerol/methanol mixtures by iterative fitting of NMR-derived 13C-labelling data from proteinogenic amino acids. N Biotechnol. 2014;31: 120–132. doi:10.1016/j.nbt.2013.06.007

3. Schellenberger J, Que R, Fleming RMT, Thiele I, Orth JD, Feist AM, et al. Quantitative prediction of cellular metabolism with constraint-based models: the COBRA Toolbox v2.0. Nat Protoc. 2011;6: 1290–307. doi:10.1038/nprot.2011.308
